# Uniportal Robotic Lobectomy and Lymphadenectomy for Invasive Lung Cancer: A Novel Approach and Perioperative Outcomes

**DOI:** 10.1111/1759-7714.15500

**Published:** 2024-11-27

**Authors:** Shengcheng Lin, Xiangyang Yu, Yafei Xu, Yu Xin, Jie He, Zhentao Yu, Hongbo Zhao, Chenglin Yang, Kai Ma

**Affiliations:** ^1^ Department of Thoracic Surgery, National Cancer Center/National Clinical Research Center for Cancer/Cancer Hospital and Shenzhen Hospital Chinese Academy of Medical Sciences and Peking Union Medical College Shenzhen Guangdong China; ^2^ Department of Anesthesiology, National Cancer Center/National Clinical Research Center for Cancer/Cancer Hospital and Shenzhen Hospital Chinese Academy of Medical Sciences and Peking Union Medical College Shenzhen Guangdong China

**Keywords:** minimally invasive surgery, non‐small cell lung cancer (NSCLC), robot‐assisted thoracoscopic surgery (RATS), uniportal

## Abstract

**Background:**

Multiport robot‐assisted thoracoscopic surgery (mRATS) has been comprehensively evaluated for its clinical efficacy in numerous studies. Nevertheless, the safety and feasibility of uniportal robotic lobectomy and lymphadenectomy require further validation.

**Methods:**

The clinical data of 34 consecutive patients with lung cancer who underwent improved uniportal robotic‐assisted thoracoscopic surgery (uRATS) at our hospital between November 2023 and June 2024 were reviewed retrospectively. Camera‐centered uRATS was conducted using the da Vinci Surgical Xi system (Intuitive Surgical Inc., 1266 Kifer Road, Sunnyvale, CA 94086, USA). Descriptive statistics are expressed as numbers with percentages for categorical data or medians (ranges) or means with standard deviations for continuous data.

**Results:**

Improved uRATS lobectomy and lymphadenectomy were conducted in 34 patients with postoperative pathology‐diagnosed invasive lung cancer. Among the patients, the median number of lymph nodes dissected was 24.5 (range 10–42), and the median number of stations with lymph nodes dissected was 8 (range 6–11). The median durations of the operation and the uRATS procedure were 200 min (range, 142–330 min) and 140 min (range, 80–242 min), and the median intraoperative blood loss volume was 20 mL (range, 10–100 mL), respectively. All postoperative complications, including pneumonia (2/34, 5.8%), air leakage > 5 days (2/34, 5.8%), prolonged wound healing (1/34, 2.9%), and arrhythmia (1/34, 2.9%), were graded as Clavien–Dindo grades I–II. There were no cases of wound infection or postoperative 30‐day mortality.

**Conclusion:**

The safety and feasibility of uRATS lobectomy and lymphadenectomy using the da Vinci Surgical Xi system have been preliminarily validated.

## Graphical abstract

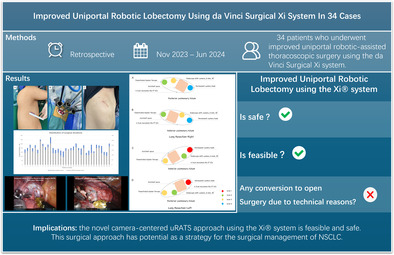

**Abbreviations**
BMI: body mass index
ECOG: Eastern Cooperative Oncology Group
FEV1: forced expiratory volume in 1 s
FVC: forced vital capacity
ICS: intercostal space
LVI: lymphovascular invasion
mRATS: multiport robotic‐assisted thoracoscopic surgery
mVATS: multiport video‐assisted thoracoscopic surgery
NSCLC: non‐small cell lung cancer
PET/CT scan: positron emission tomography/computed tomography scan
RATS: robot‐assisted thoracoscopic surgery
STAS: spread through air spaces
TNM staging system: tumor node metastasis staging system
uRATS: uniportal robotic‐assisted thoracoscopic surgery
VPI: visceral pleural invasion
WHO: World Health Organization

## 1 Introduction

Robot‐assisted thoracoscopic surgery (RATS) was first reported in 2002 by Melfi et al. [1]. Since then, RATS has become the popular technique in thoracoscopic surgery in several centers worldwide [2, 3] owing to the following advantages: visualization of the area of operation, precision, maneuverability, and tremor filtration. Currently, the most complex resections, such as pneumonectomy [4], bronchovascular sleeves [5, 6], and carinal reconstructions, can be achieved with RATS [7]. Currently, multiport robot‐assisted thoracic surgery (mRATS) with 3 or 4 ports and 1 or 2 assistive incisions remains the standard surgical treatment option among most thoracic surgeons [8]. Thoracoscopic pulmonary surgery and uniportal video‐assisted thoracoscopic surgery (uVATS) have potential advantages over multiport video‐assisted thoracoscopic surgery (mVATS), such as less postoperative pain [9] and better visualization of the sagittal view [7]. Whether the advantages of uniportal robot‐assisted thoracoscopic surgery (uRATS) are similar to those of mRATS warrants investigation.

Recently introduced in 2021, uRATS allows the use of three arms through a single incision using the da Vinci Xi system, and its potential in thoracic surgery has been elucidated [10]. Despite its recent implementation and endorsement in several centers, this technique was already used in all types of surgeries, including carinal sleeve resection [11], pneumonectomy [12], lobectomy [13], and segmentectomy [14]. The potential advantages of this approach are shorter docking times, faster port placement, seemingly lower cost, less pain, and greater manageability of emergencies. However, studies on the use of uRATS in high‐volume centers are lacking, so studies on whether this technique can be replicated and improved are needed.

In this single‐center retrospective study, we report our initial experience in performing uRATS lobectomy and lymphadenectomy for the treatment of 34 cases of non‐small lung cancer (NSCLC), with a focus on feasibility, safety, surgical technique, and early postoperative outcomes. The clinical efficacy of improved uRATS was preliminarily validated.

## 2 Methods

This single‐institution retrospective study was approved by the Cancer Hospital and Shenzhen Hospital, the Chinese Academy of Medical Sciences, and the Peking Union Medical College Institutional Review Board of Clinical Research (no. YW2024‐1‐1). The study was conducted in accordance with the tenets of the Declaration of Helsinki (as revised in 2013). Owing to the retrospective study design, the requirement for informed consent was waived.

### 2.1 Patients

Uniportal robotic‐assisted thoracoscopic surgery was initially performed for the diagnosis and treatment of invasive lung cancer at our hospital, starting on November 2, 2023, and ending on June 25, 2024. The data of 35 consecutively selected patients were included in this study retrospectively. One patient underwent conversion to thoracotomy and left pneumonectomy due to vascular and tracheal invasion instead of uRATS. All included patients underwent preoperative examinations, including blood tests, imaging, and functional tests, including positron emission tomography (PET)‐computed tomography (CT), spirometry, echocardiography, magnetic resonance imaging (MRI)/CT of the brain with intravenous contrast, and bronchoscopy/endobronchial ultrasound transbronchial needle aspiration for diagnosis and staging. The clinicopathological characteristics of these patients were subsequently collected, including baseline information and pathological results. The histological subtypes were classified in accordance with the 2021 World Health Organization (WHO) classification system [15], and the pathological stages were evaluated using the eighth edition of the tumor node metastasis (TNM) staging system [16].

### 2.2 Improved uRATS Technique

**FIGURE 1:**
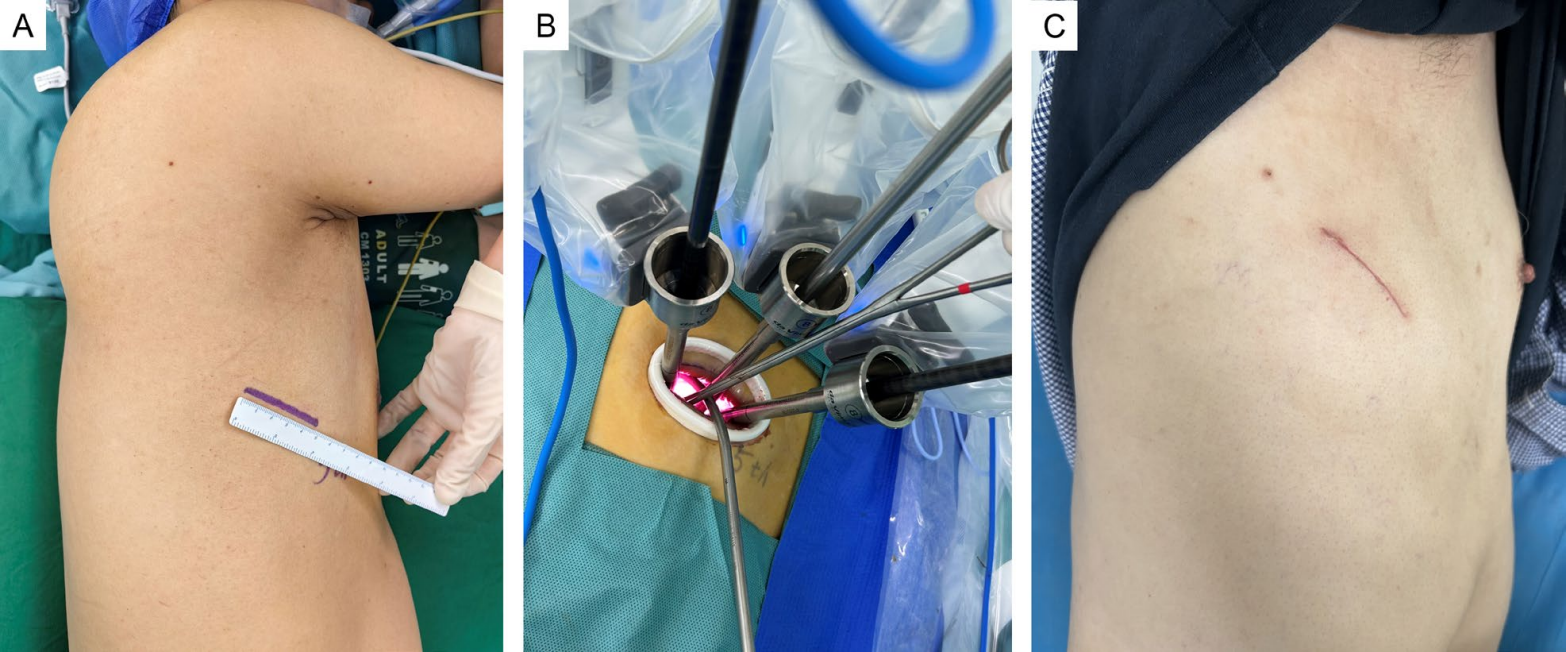
Port design for uniportal RATS. (A) A 5 cm utility incision was made in the fifth ICS during the operation. (B) Robotic arm placement for right lung resection: Fenestrated bipolar forceps were usually placed at the upper edge of the incision via arm 2, and an endoscope with a camera was subsequently placed close to arm 2 via arm 3. A permanent cautery hook was then placed close to the lower edge of the incision by arm 4. The spaces between arms 3 and 4 also allow the assistant to employ oval forceps or suction devices for posterior pulmonary hilum procedures, while space is available between arms 2 and 3 for anterior pulmonary hilum procedures. (C) Close‐up of a surgical scar on the chest of a 43‐year‐old male patient following uniportal RATS lobectomy. ICS, intercostal space; RATS, robotic‐assisted thoracoscopic surgery.

**FIGURE 2:**
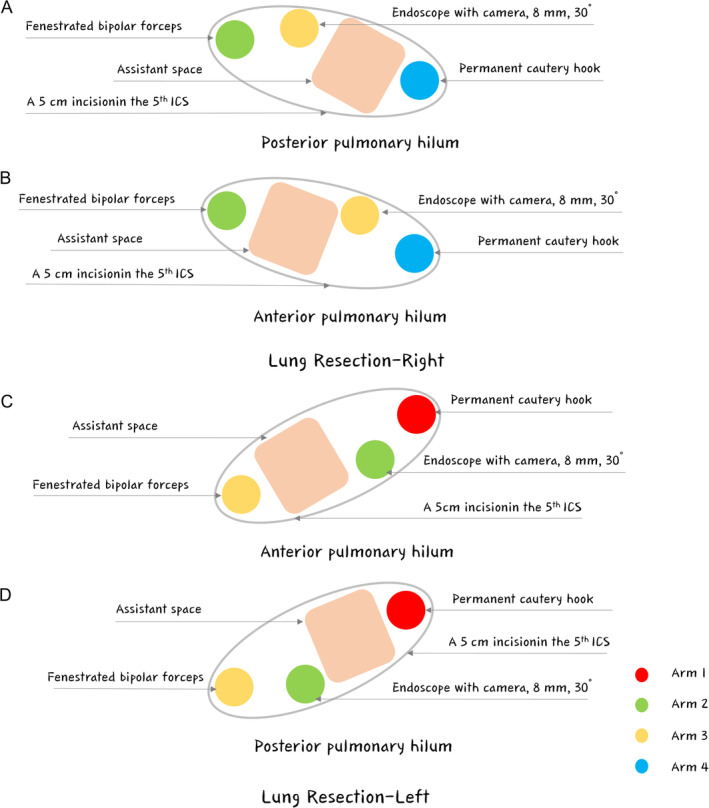
Landscape with instrument placement. (A, B) For right lung resection, fenestrated bipolar forceps were usually placed at the upper edge of the incision by arm 2, and an endoscope with a camera was subsequently placed close to arm 2 by arm 3. The permanent cautery hook was then placed close to the lower edge of the incision by arm 4. The spaces between arms 3 and 4 also allow the assistant to employ oval forceps or suction devices for posterior pulmonary hilum procedures, whereas space is available between arms 2 and 3 for anterior pulmonary hilum procedures. (C, D) For left lung resection, a permanent cautery hook was then placed close to the upper edge of the incision by arm 1, and an endoscope with a camera was subsequently placed close to arm 1 using arm 2. The fenestrated bipolar forceps were usually placed at the lower edge of the incision by arm 3. The spaces between arms 1 and 2 also allow the assistant to employ oval forceps or suction devices for posterior pulmonary hilum procedures, whereas space between arms 2 and 3 is available for anterior pulmonary hilum procedures.

Improved uRATS was conducted using the da Vinci Surgical Xi system (Intuitive Surgical Inc., 1266 Kifer Road, Sunnyvale, CA 94086, USA), which has been certified and applied at our institution since February 2022. All surgical procedures were conducted by the same surgeon. A 4‐ to 5‐cm utility incision was customarily placed in the 5th intercostal space (ICS) between the middle and posterior axillary lines in patients in the FLEX position, enlarging it depending on the surgical procedure (Figure 1A). According to the potential for malignancy (including the size of the nodule, radiographic features, rate of growth, age of the patient, and presence of risk factors), diagnostic intraoperative wedge resection and frozen section were recommended during uVATS for indeterminate peripheral lung nodules. Extra robot‐related costs were waived when the frozen section indicated that the nodule was benign. Patients with NSCLC underwent uRATS utilizing the Da Vinci system. Once the center point of the da Vinci system aligns with the incision, the robotic arm subsequently rotates perpendicularly to the incision (Figure 1B). The procedure was performed using three robotic arms (30° robotic camera, robotic instruments, and robotic staplers (optional)) and uVATS‐type instruments (graspers with subxiphoid lengths, dissectors, staples (optional), and long‐curved suctions (subxiphoid instruments)). Three robotic arms (1, 2, and 3 arms (for left lung resection) or 2, 3, and 4 arms (for right lung resection)) are usually selected during the procedure (Figure 2A–D). A Fenestrated Bipolar Forceps and Permanent Cautery Hook are typically used on the other two arms. Other optional instruments are the Maryland bipolar forceps and Harmonic ACE curved shears.

A novel camera‐centered approach to uRATS lobectomy and lymphadenectomy has been adopted, which is completely different from the reported camera‐backed robotic arm arrangement [10]. For right lung resection, fenestrated bipolar forceps were usually placed on the upper edge of the incision by arm 2, and an endoscope with a camera was subsequently placed close to arm 2 by arm 3. The permanent cautery hook was then placed on the lower edge of the incision by arm 4. The spaces between arms 3 and 4 allow the assistant to employ oval forceps or suction devices for posterior pulmonary hilum procedures, and space is available between arms 2 and 3 for anterior pulmonary hilum procedures (Figure 1A–D and Video S4). For left lung resection, a permanent cautery hook was then placed on the upper edge of the incision by arm 1, and an endoscope with a camera was subsequently placed close to arm 1 by arm 2. Fenestrated bipolar forceps were usually placed on the lower edge of the incision by arm 3. The spaces between arms 1 and 2 also allow the assistant to employ oval forceps or suction devices for posterior pulmonary hilum procedures, and space is available between arms 2 and 3 for anterior pulmonary hilum procedures (Figure 1A–D and Video S5). During the procedure, the hilar structures are isolated, and the lymph nodes are removed using robotic arms (Videos [Link], [Link], [Link]), whereas the hilar structures and fissures are divided consecutively using a VATS stapler, which reaches directly into the thorax and adjusts to the position of the hilar structures (Videos S4 and S5). A chest drain tube was placed at the posterior edge of the incision. The incision was closed in the same way as with uVATS (Figure 1C). The chest drain tube was removed if no air leakage was present and if the drainage volume was less than 200 mL within 24 h. Prolonged air leaks were defined if the air leak lasted more than 5 days. All postoperative complications were recorded and graded according to the Clavien–Dindo classification [17].

### 2.3 Statistical Analysis

Descriptive statistics are expressed as numbers with percentages for categorical data or medians (ranges) or means with standard deviations for continuous data. All the statistical analyses were performed using SPSS (version 26, IBM Corp., Armonk, NY, USA), and all the graphs were created using GraphPad Prism (version 9.0, Dotmatics, CA, USA). All the *p* values were two‐sided, and a *p* value < 0.05 indicated statistical significance.

## 3 Results

### 3.1 Clinicopathological Characteristics

Improved uRATS lobectomy and lymphadenectomy were performed in 34 patients (Table 1). Our cohort was comprised of 16 males (16/34, 47.1%) and 18 females (18/34, 52.9%), with middle‐aged and elderly patients accounting for the greatest percentage (median, 61.0 years; range, 37–79 years). The median body mass index (BMI) of all the patients was 21.6 kg/m^2^ (range, 16.7–26.8 kg/m^2^). Only seven patients (7/34, 20.6%) were current smokers (6/34, 17.6%) or ever smoked (1/34, 2.9%). The median tumor size was 24.5 mm (range, 14–75 mm). All patients had Eastern Cooperative Oncology Group (ECOG) scores ranging from 0 (31/34, 91.2%) to 1 (3/34, 8.8%). Nodes were located on the right lower (12/34, 35.2%), upper (6/34, 17.6%), and middle (4/34, 11.8%) lobes and on the left upper (8/34, 23.5%) and lower (7/34, 20.6%) lobes. One patient (1/34, 2.9%) had nodules in both the right upper and right lower lobes; one patient (1/34, 2.9%) had nodules in both the right middle and right lower lobes; and one patient (1/34, 2.9%) had nodules in both the right lower and left upper lobes. Overall, pulmonary function was favorable in these patients, with a median forced vital capacity (FVC) of 93.0% (range 70.7%–134.1%) and a forced expiratory volume in 1 s (FEV1) of 90.6% (range 52.5%–128.4%). Thirty‐four patients had 22 comorbidities, including hypertension (7/34, 20.6%), diabetes mellitus (3/34, 8.8%), arrhythmia (3/34, 8.8%), coronary artery disease (2/34, 5.9%), gallstones (2/34, 5.9%), cerebral infarction (2/34, 5.9%), tuberculosis (1/34, 2.9%), hyperlipoidemia (1/34, 2.9%), and hepatitis B (1/34, 2.9%). No patient had extrathoracic malignancy or chronic obstructive pulmonary disease. Overall, according to the TNM staging system [16], nineteen patients (55.9%), twelve patients (35.3%), two patients (5.9%), and one patient (2.9%) were evaluated for clinical tumor stages I, 2, 3, and 4, respectively. One patient (1/34, 2.9%) and three patients (3/34, 8.8%) were clinically diagnosed with N1 and N2 by positron emission tomography (PET)/CT scan, and two patients (3/34, 8.8%) were pathologically diagnosed with N2 by endobronchial ultrasound‐transbronchial fine needle aspiration.

**TABLE 1:** Clinicopathological characteristics.

Variable^a^	Values (*N* = 34)
Age, year	61 (37–79) 59.5 ± 10.9
Sex	
Male	16 (47.1)
Female	18 (52.9)
BMI, kg/m^2^	21.6 (16.7–26.8) 21.8 ± 2.2
Smoking	
Current or ever	7 (20.6)
Never	27 (79.4)
Nodule size, mm	24.5 (14–75) 24.6 ± 17.8
ECOG performance status	
0	31 (91.2)
1	3 (8.8)
2	0 (0)
3	0 (0)
Nodule location (lobe)^b^	
Right upper	6 (17.6)
Right middle	4 (11.8)
Right lower	12 (35.2)
Left upper	8 (23.5)
Left lower	7 (20.6)
Pulmonary function tests, %	
FVC	93.0 (70.7–134.1) 95.4 ± 16.2
FEV1	90.6 (52.5–128.4) 89.1 ± 16.3
Comorbidity (22 comorbidities in 34 patients)	
Extrathoracic malignancy	0 (0)
Hypertension	7 (20.6)
Diabetes mellitus	3 (8.8)
Coronary artery disease	1 (2.9)
COPD	0 (0)
Others (arrhythmia, hepatitis B, etc.)	14 (41.2)
PET scan	16 (47.1)
Invasive mediastinal staging	
Endobronchial ultrasound	10 (29.4)
Mediastinoscopy	0 (0.00)
Induction treatment	
Immunochemotherapy	2 (5.9)
Targeted therapy	4 (11.8)
Clinical T‐stage	
1	19 (55.9)
2	12 (35.3)
3	2 (5.9)
4	1 (2.9)
Clinical N‐stage	
0	30 (88.2)
1	1 (2.9)
2	3 (8.8)

Abbreviations: BMI, body mass index; COPD, chronic obstructive pulmonary disease; ECOG, Eastern Cooperative Oncology Group; FEV1, forced expiratory volume in 1 s; FVC, forced vital capacity; PET/CT scan, positron emission tomography/computed tomography scan.

^a^ Descriptive statistics are expressed as numbers with percentages for categorical data or medians (ranges) or means and standard deviations for continuous data.

^b^ One patient had nodules in both the right upper and right lower lobes; one patient had nodules in both the right middle and right lower lobes; and one patient had nodules in both the right lower and left upper lobes.

### 3.2 Perioperative Outcomes

**FIGURE 3:**
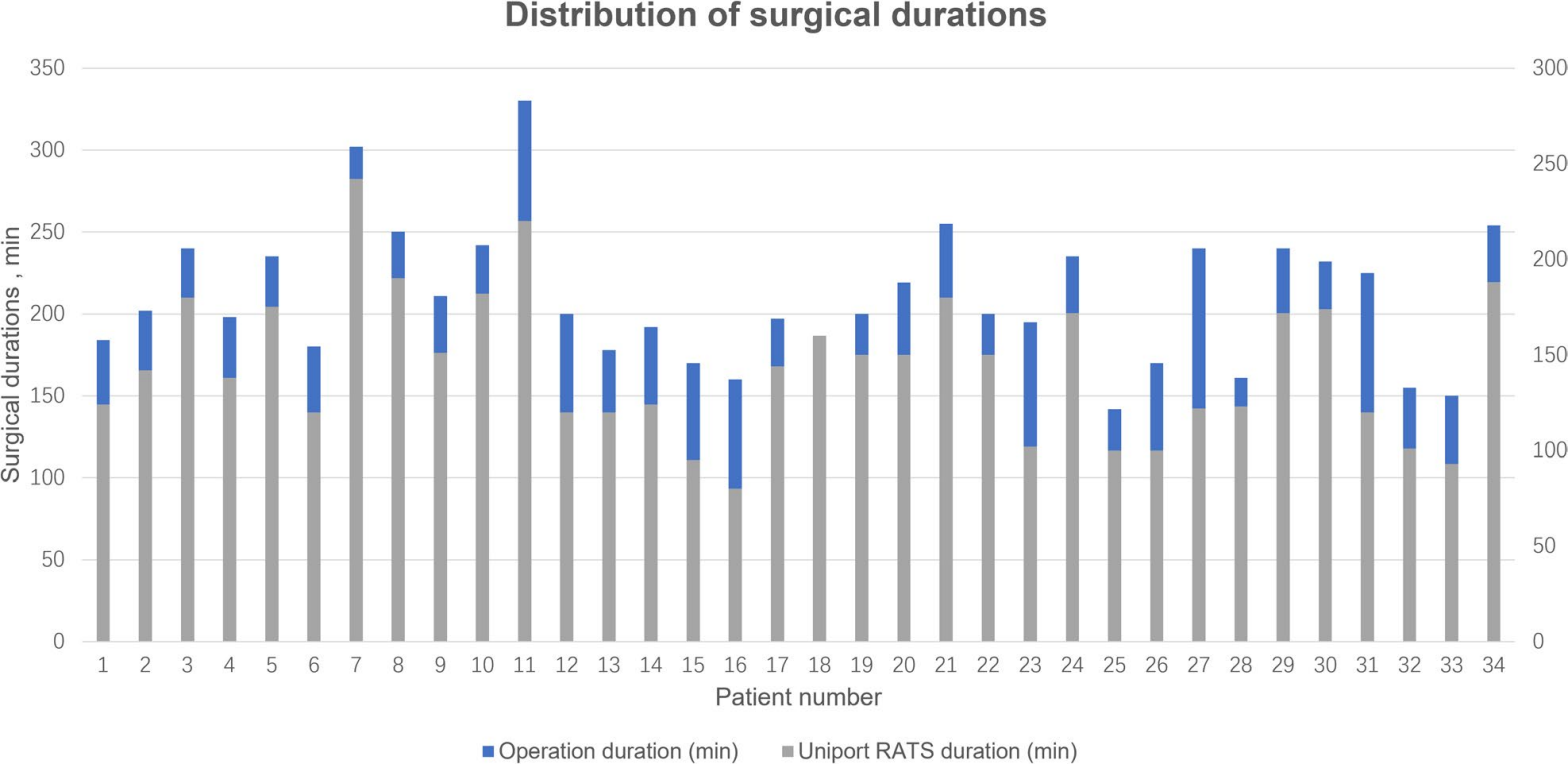
Distribution of surgical durations. RATS, robot‐assisted thoracoscopic surgery.

Lobectomy was performed for all patients (Table 2), including those who underwent right upper (6/34, 17.6%), right middle (4/34, 11.8%), right lower (10/34, 29.4%), left upper (6/34, 17.6%), and left lower lobectomy (8/34, 23.5%). Four patients underwent combined lobectomy with wedge resection (4/34, 11.8%), and 1 underwent combined right upper and middle lobectomy (1/34, 2.9%). Among the patients who underwent lymph node dissection (34 patients), the median number of lymph nodes dissected was 24.5 (range 10–42), and the median number of stations with lymph nodes dissected was 8 (range 6–11). For the entire cohort, the median operation duration and duration of the uRATS procedure were 200 min (range 142–330 min) and 140 min (range 80–242 min), respectively (Figure 3), with a median intraoperative blood loss volume of 20 mL (range 10–100 mL). There were no reoperations. The median durations of postoperative hospitalization and chest tube placement were 6 days (range, 4–17 days) and 4 days (range, 3–14 days), respectively. Postoperative complications included pneumonia (2/34, 5.8%), air leakage for > 5 days (2/34, 5.8%), prolonged wound healing (1/34, 2.9%), and arrhythmia (1/34, 2.9%), all of which were considered Clavien–Dindo grades I–II, and none of the patients experienced grades III–IV complications. There were no cases of wound infection or postoperative 30‐day mortality.

**TABLE 2:** Perioperative outcomes.

Variable^a^	Values (*N* = 34)
Resection methods^b^	
Lobectomy	34 (100)
Wedge resection	4 (11.8)
Lymph nodes dissected, No.	24.5 (10–42) 24.8 ± 7.1
Lymph nodes stations dissected, No.	8.0 (6–11) 8.1 ± 1.3
Operation duration (min)	200 [142–330] 209.5 ± 42.0
Uniport RATS duration (min)	140 [80–242] 141.3 ± 35.9
Intraoperative blood loss (mL)	20 [10–100] 25.6 ± 17.3
Reoperation	0 (0)
Length of postoperative stay (days)	6 [4–17] 7.4 ± 3.3
Drainage duration (days)	4 [3–14] 4.9 ± 2.1
Drainage volume (mL)	746 [2–1760] 797.3 ± 405.8
Postoperative complications^c^	
Pneumonia	2 (5.8)
Air leak > 5 days	2 (5.8)
Postoperative bleeding	0 (0)
Arrhythmia	1 (2.9)
Blood transfusion	0 (0)
Prolonged wound healing	1 (2.9)
Clavien–Dindo I–II	6 (17.6)
Clavien–Dindo III–IV	0 (0)
Morbidity within 30 days	0 (0.0)
Mortality within 30 days	0 (0.0)

Abbreviations: RATS, robot‐assisted thoracoscopic surgery; VATS, video‐assisted thoracoscopic surgery.

^a^ Descriptive statistics are expressed as numbers with percentages for categorical data or medians (ranges) or means with standard deviations for continuous data.

^b^ 4 patients underwent combined lobectomy with wedge resection, and 1 patient underwent combined right upper and middle lobectomy.

^c^ Postoperative complications were evaluated in accordance with the Clavien–Dindo classification.

### 3.3 Pathologic Outcomes

Concerning the pathological outcomes, most of the patients (30/34, 88.2%) were diagnosed with a solitary lesion, and adenocarcinoma being the prominent histological subtype (28/34, 82.4%) (Table 3). Four patients had simultaneous multiple primary lung adenocarcinomas (4/34, 11.8%), 1 patient had squamous cell carcinoma (1/34, 2.9%), and 1 patient had lymphoepithelioma‐like carcinoma (1/34, 2.9%). There was no adenocarcinoma in situ or microinvasive adenocarcinoma. According to the WHO classification [15], 2 (5.8%), 15 (44.1%), and 19 (55.9%) specimens were classified as grades I, II, and III, respectively. Sixteen (47.1%), 8 (43.3%), and 8 (43.3%) samples were also evaluated for the presence of “spread through air spaces (STAS),” “visceral pleural invasion (VPI),” and “lymphovascular invasion (LVI),” respectively, which are also important indicators of prognosis. On the basis of the TNM staging system [16], 1 (2.9%), 20 (58.8%), 7 (20.6%), and 6 (17.6%) patients had stages 0, I, II, and III disease, respectively. One of the six patients who received neoadjuvant therapy experienced complete pathologic remission, and three were downstaged.

**TABLE 3:** Pathologic outcomes.

Variable^a^	Values (*N* = 34)
Histological classification, *n* (%)	
Adenocarcinoma	32 (94.1)
AIS or MIA	0 (0)
Invasive adenocarcinoma^b^	32 (94.1)
Grade I	2 (5.8)
Grade II	15 (44.1)
Grade III	19 (55.9)
Squamous cell carcinoma	1 (2.9)
Others (pleomorphic, mixed type, etc.)	1 (2.9)
Pathological T stage^c^	
pTis	0 (0)
pT1a (mi)	0 (0)
pT1a	1 (2.9)
pT1b	6 (17.6)
pT1c	9 (26.5)
pT2a	10 (29.4)
pT2b	5 (14.7)
pT3	2 (5.8)
NA^c^	1 (2.9)
Pathological N stage^d^	
pN0	27 (79.4)
pN1	1 (2.9)
pN2	6 (17.6)
NA^d^	0 (0)
Pathological TNM stage^d^	
0	1 (2.9)
IA1	1 (2.9)
IA2	4 (11.8)
IA3	8 (23.5)
IB	7 (20.6)
IIA	4 (11.8)
IIB	3 (8.8)
IIIA	6 (17.6)

Abbreviations: AIS, adenocarcinoma in situ; MIA, microinvasive adenocarcinoma; TNM stage, tumor‐nodes‐metastasis stage; WHO, World Health Organization.

^a^ Descriptive statistics are expressed as numbers with percentages for categorical data or medians (ranges) or means and standard deviations for continuous data.

^b^ All invasive adenocarcinomas were graded according to the WHO classification.

^c^ Pathological stage was classified according to the eighth edition of the TNM classification.

^d^ One patient exhibiting a pathological complete response to induction treatment for lung cancer.

## 4 Discussion

Surgical resection remains the preferred treatment for early‐stage NSCLC [18]. Compared with conventional thoracotomy, minimally invasive thoracic surgery is recognized as a safe and effective surgical option for the treatment of this group of lung cancer patients because of improvements in endoscopic instruments and surgical technologies [13]. In particular, thoracoscopic lung resection has been widely performed in the treatment of lung cancer because of its ability to minimize postoperative pain, shorten the hospitalization time, increase the rate of return to work, decrease the incidence of pulmonary complications, and decrease lung function damage without affecting long‐term survival [9, 13, 19]. In recent years, multiport robotic thoracoscopic technology has rapidly evolved, and it has been widely applied in lung resection surgery, including the most complex resections [5], because of its stability, maneuverability, and accuracy [2]. In this study, we preliminarily verified that a novel camera‐centered approach involving uRATS lobectomy and lymphadenectomy using the da Vinci Xi system is a feasible and safe technique for lung resection by surgeons experienced in managing NSCLC.

Over the last two decades, minimally invasive thoracoscopic surgery has evolved from thoracoscopic approaches using three to four ports to single‐port approaches [13] and from conventional thoracoscopic techniques with VATS to robotic thoracoscopic techniques with RATS [2]. Compared with conventional multiport (three‐ to four‐port) VATS, thoracoscopic VATS is associated with less postoperative pain, a lower rate of residual paralysis, and shorter hospital stays [9, 19]. In terms of thoracoscopic techniques, comparisons of VATS and RATS present contradictory conclusions. Several studies have revealed that RATS for major lung resection reduces bleeding, shortens the hospitalization time, decreases retraction rates, and improves lymph node assessments compared with VATS, especially in NSCLC patients with pathologically N2 disease [2, 20]. Nevertheless, in other studies, surgical outcomes, including perioperative complications, length of hospitalization, 30‐day mortality, and nodal upstaging, have shown that robotic lobectomy is equivalent to VATS [21]. Taken together, the current evidence suggests that robotic lung surgery has a therapeutic efficacy comparable to that of VATS, with slight disadvantages in terms of the operative time and cost of instrumentation. Despite its acceptance, there are still some issues regarding the robotic thoracoscopic approach, including the use of endoscopic instruments versus conventional instruments and the number of incisions.

Uniportal robotic‐assisted thoracoscopic surgery procedures were initially performed in 2021 and involved the use of a hybrid method involving the da Vinci Xi robotic system with the VATS stapler, and its feasibility was preliminarily validated [10]. Subsequently, Gonzalez‐Rivas et al. described in detail the early experience with uRATS using a robotic stapler, and this approach was also called pure uniportal robotic surgery [22]. Both are not recommended for use with the Da Vinci XI robotic system. Furthermore, 3 robotic arms were precisely placed through an intercostal incision, and then lung resections were performed [8]. Given the similarity of this technique to uVATS, it presents the possibility for thoracic surgeons experienced in minimally invasive techniques to switch to a robot‐assisted approach. Currently, uRATS for various major lung resections, such as lobectomy [13], segmentectomy [14], pneumonectomy [12], and sleeve resection [7], has been successfully implemented in several centers. It is cost‐effective in oncology and economics and therefore provides patients with better outcomes than conventional methods do [13]. Preliminary results from a European multicenter study of 101 uniportal cases revealed that uRATS is feasible and safe for anatomical lung resection and is comparable to the multiportal conventional approach in terms of surgical outcomes [23]. A German small sample study similarly demonstrated that the uRATS/uVATS results were comparable, highlighting the technology's potential and applicability [24].

**FIGURE 4:**
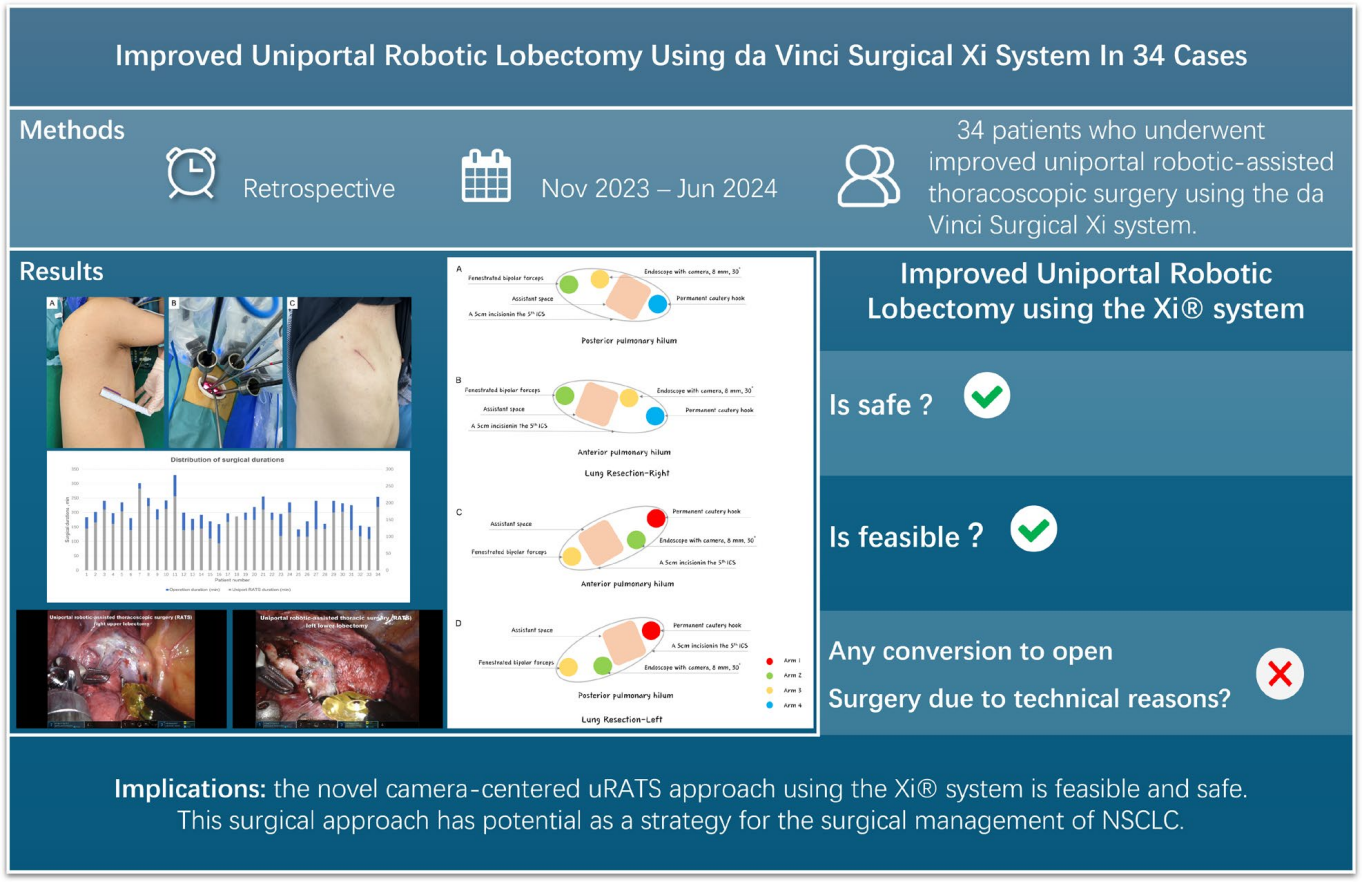
Improved uniportal robotic lobectomy using da Vinci Surgical Xi system. uRATS, uniportal robot‐assisted thoracoscopic surgery.

To date, a total of 35 patients are scheduled for uniportal robotic lobectomy and lymphadenectomy at our center, and all patients evaluated for surgery are clinically diagnosed with invasive NSCLC. Several patients had locally advanced disease, and only one patient was converted to open left pneumonectomy due to vascular and tracheal invasions instead of the use of the uRATS surgical maneuver. The median duration was 200 min for total surgery and 140 min for the uRATS procedures (Figure 3). The duration of routine VATS seems to be longer, and possible factors include preparation of the robotic arms, wedge resection for definitive pathology, the release of extensive thoracic adhesions, intraoperative removal, adjustment of the robotic arm, and unsatisfactory lung collapse. Currently, relatively advanced surgical procedures, uRATS, have been established; furthermore, a relatively mature (Figure 3, Videos [Link], [Link], [Link], [Link], [Link]), which are routinely used in our center for major lung resection. Highlights from our technology are summarized in Figure 4. We consider that this technology has several advantages. First, diagnostic thoracoscopic wedge resection for patients without preoperative pathologic diagnosis can be conducted, which avoids the costly financial expenses of robotic use in benign patients. Second, unlike previous reports in which the camera was placed at the back of the incision [13, 25], we positioned the camera of the robotic arm in the center of the incision (Figures 1 and 2). This novel approach provides more maneuvering space for the two robotic arms, thus avoiding interference. Third, systematic lymph node dissection was performed, and clearance was satisfactory. The median number of dissected lymph nodes was 24.5 and the median number of dissected lymph node dissection stations was 8 (Video S3). Finally, two suction instruments, a long, curved suction device (subxiphoid instruments) and a normal suction device, were used to balance suctioning and improve the visualization of the anatomical position, thus providing the operator with more space to maneuver and reduce the incidence of robotic arm interference.

Several observations about this technique might be made on the basis of our initial experience with uRATS. The incision is typically located between the 5th intercostal space from the mid‐axillary line to the posterior axillary line, thus allowing sufficient space for two operating robotic arms. Additionally, the camera of the robotic arm is placed as close to the incision as possible to minimize the depth of insertion into the thoracic cavity, whereas the other two robotic arms remain located at different depths in the thoracic cavity, thus further minimizing the interference of the trocars outside the patient's body. Furthermore, the advantages of uRATS can be illustrated easily in clinical practice since the incision is made in both the anterior and posterior hilum, providing thorough resection of both the hilar and mediastinal lymph nodes (Videos [Link], [Link], [Link], [Link], [Link]). After the hilum is isolated, the stapler can be placed without difficulty. One to two robotic arms can be withdrawn if maneuvering is difficult.

Several limitations are observed in our study. First, since it was a single‐center retrospective study, the study period was short, and the sample size was limited. Second, the advantages and disadvantages of the uRATS have not been compared with those of the uVATS or mRATS. Finally, a survival outcome of uRATS is needed to illustrate the oncologic treatment effect.

## 5 Conclusions

According to the characterization of the early outcomes of this modality, the novel uRATS approach is feasible and safe, and its clinical efficacy has been shown preliminarily. This surgical approach has potential as a strategy for the surgical management of NSCLC.

## Author Contributions

All the authors read and approved the final version of the manuscript. Conceptualization: K.M., S.L., X.Y. and Y.X. Methodology: K.M., S.L., X.Y. and Y.X. Project administration: K.M. and Z.Y. Resources: K.M., Z.Y. and S.L. Data curation: K.M., S.L., X.Y., Y.X., Y.X., J.H., Z.Y., H.Z. and C.Y. Formal analysis: K.M., S.L., X.Y., Y.X., Y.X., J.H., Z.Y., H.Z. and C.Y. Writing – original draft preparation: S.L., X.Y. and Y.X. Writing – review and editing: K.M., S.L., X.Y., Y.X., Y.X., J.H., Z.Y., H.Z. and C.Y.

## Ethics Statement

The study was approved by the Clinical Research Institute Review Committee of the Chinese Academy of Medical Sciences and Peking Union Medical College Cancer Hospital and Shenzhen Hospital (Shenzhen, China) on February 1, 2024 (approval no. YW2024‐1‐1), and it followed the Declaration of Helsinki. Written informed consent was obtained from all the patients.

## Conflicts of Interest

The authors declare no conflicts of interest.

## Supporting information


**Video S1** Improved uniportal RATS lymph node dissection (stations 2 and 4). RATS, robot‐assisted thoracoscopic surgery.

**Video S2** Improved uniportal RATS lymph node dissection (stations 5 and 6). RATS, robot‐assisted thoracoscopic surgery.

**Video S3** Improved uniportal RATS lymph node dissection (station 7). RATS, robot‐assisted thoracoscopic surgery.

**Video S4** Improved uniportal RATS right upper lobectomy. RATS, robot‐assisted thoracoscopic surgery.

**Video S5** Improved uniportal RATS left lower lobectomy. RATS, robot‐assisted thoracoscopic surgery.

## Data Availability

Data are available on request to the authors.
